# Alpha-tocopherol enhances spermatogonial stem cell proliferation and restores mouse spermatogenesis by up-regulating BMI1

**DOI:** 10.3389/fnut.2023.1141964

**Published:** 2023-04-17

**Authors:** Mei Peng, Jintao Wu, Weifan Wang, Tianlong Liao, Simeng Xu, Di Xiao, Zuping He, Xiaoping Yang

**Affiliations:** Key Laboratory of Study and Discovery of Small Targeted Molecules of Hunan Province, The Key Laboratory of Model Animals and Stem Cell Biology in Hunan Province, Key Laboratory of Chemical Biology & Traditional Chinese Medicine Research of Ministry of Education, Key Laboratory of Protein Chemistry and Developmental Biology of Fish of Ministry of Education, Department of Pharmacy, Hunan Normal University, Changsha, Hunan, China

**Keywords:** BMI1, spermatogonial stem cells, proliferation and DNA synthesis, DNA damage, sperm count and malformation, α-tocopherol

## Abstract

**Purpose:**

Spermatogonial stem cells (SSCs) are essential for maintaining reproductive function in males. *B*-lymphoma Mo-MLV insertion region 1 (BMI1) is a vital transcription repressor that regulates cell proliferation and differentiation. However, little is known about the role of BMI1 in mediating the fate of mammalian SSCs and in male reproduction. This study investigated whether BMI1 is essential for male reproduction and the role of alpha-tocopherol (α-tocopherol), a protective agent for male fertility, as a modulator of BMI1 both *in vitro* and *in vivo*.

**Methods:**

Methyl thiazolyl tetrazolium (MTT) and 5-ethynyl-2′-deoxyuridine (EDU) assays were used to assess the effect of BMI1 on the proliferative ability of the mouse SSC line C18-4. Real-time polymerase chain reaction (PCR), western blotting, and immunofluorescence were applied to investigate changes in the mRNA and protein expression levels of BMI1. Male mice were used to investigate the effect of α-tocopherol and a BMI1 inhibitor on reproduction-associated functionality *in vivo*.

**Results:**

Analysis revealed that BMI1 was expressed at high levels in testicular tissues and spermatogonia in mice. The silencing of BMI1 inhibited the proliferation of SSCs and DNA synthesis and enhanced the levels of γ-H2AX. α-tocopherol enhanced the proliferation and DNA synthesis of C18-4 cells, and increased the levels of BMI1. Notably, α-tocopherol rescued the inhibition of cell proliferation and DNA damage in C18-4 cells caused by the silencing of BMI1. Furthermore, α-tocopherol restored sperm count (Ctrl vs. PTC-209, *p* = 0.0034; Ctrl vs. PTC-209 + α-tocopherol, *p* = 0.7293) and normalized sperm malformation such as broken heads, irregular heads, lost and curled tails *in vivo*, as demonstrated by its antagonism with the BMI1 inhibitor PTC-209.

**Conclusion:**

Analysis demonstrated that α-tocopherol is a potent *in vitro* and *in vivo* modulator of BMI1, a transcription factor that plays an important role in in SSC proliferation and spermatogenesis. Our findings identify a new target and strategy for treating male infertility that deserves further pre-clinical investigation.

## Introduction

Spermatogonial stem cells (SSCs) form the basis for spermatogenesis and represent a subpopulation of type A spermatogonia in the mammalian testis (1–4). SSCs can be stimulated to develop into mature and functional spermatids that can fertilize oocytes and produce offspring. Therefore, SSCs have the potential to be utilized in the treatment of male infertility. Furthermore, SSCs are an excellent model for investigating the function and mechanisms of genetic and epigenetic regulation in adult stem cells (5–7).

B-lymphoma MO-MLV insertion region 1 (BMI1) is a component of the polycomb repressive complex 1 (PRC1) and acts as a transcription suppressor (8–10). Research has shown that BMI1 regulates stem cell self-renewal and differentiation (11–13). In addition, studies have reported that BMI1 is expressed at high levels in testicular tissue and all testicular cells. In a previous study, male mice with BMI1 deficiency were found to exhibit severe oligospermia and significantly reduced testicular volume, thus suggesting that BMI1 is necessary for maintaining normal reproductive function (14). The loss of BMI1 inhibits germ cell proliferation and induces germ cell apoptosis, thus exerting significant effect on male fertility (15). However, the precise function and mechanism of BMI1 in mediating the fate of mammalian SSCs and their role in male reproduction has yet to be elucidated.

In 1922, Evans and Bishop were the first to report that α-tocopherol, a recognized antioxidant, is required for reproductive function in rats (16). Subsequent research showed that the transport of α-tocopherol in the plasma and brains of mammals is controlled by plasma phosphatidyl transporter protein (PLTP). The loss of PLTP has been shown to reduce the levels of α-tocopherol in the mouse epididymis, thus leading to a significant reduction in sperm motility and fertilization rate *in vitro* (17). Furthermore, α-tocopherol supplementation was shown to significantly improve the viability and DNA integrity of normal and motility-low sperm after thawing (18). A randomized and double-blinded clinical trial demonstrated that the concomitant administration of DHA (22:6 N-3) and α-tocopherol led to an increase in sperm motility in asthenozoospermic men (19) while another study showed that α-tocopherol nanoemulsions reduced oxidative stress in spermatids of Red Deer, which may improve sperm quality for assisted reproductive technology (ART) (20, 21). Adequate α-tocopherol supplementation in a balanced poultry diet was shown to maintain reproductive functionality, including higher percentage of sperm concentration, total motility, progressive motility, viability, and membrane integrity in the roosters (22–24). Nevertheless, the mechanism by which α-tocopherol can maintain normal male reproductive function remains unknown.

In the present study, we revealed that BMI1 is essential for maintaining the proliferative activity of mouse SSCs and normal reproductive function in male mice. We also found that α-tocopherol can up-regulate BMI1, promote the proliferation of mouse SSCs, and maintain sperm counts and morphology in male mice. Furthermore, we applied a silencing technique *in vitro* and a specific inhibitor *in vivo* to investigate the role of BMI1 on the improvements induced by α-tocopherol. Our findings are of great significance as they provide new insight into the mechanisms underlying the proliferation of mammalian SSCs and could lead to the development of a new approach to treat male infertility.

## Materials and methods

### Reagents

Alpha-tocopherol (Sigma-Aldrich, Shanghai, China) was dissolved in dimethyl sulfoxide (DMSO) to prepare a 100 mg/mL stock solution. PTC-209, an inhibitor of BMI1 (Selleck Chemicals, Houston, TX, United States) was dissolved in DMSO to prepare a 40 mg/mL stock solution.

Immunoblotting was performed with the following antibodies: BMI1 (Abcam, Cambridge, UK. Cat. ab254253); PCNA (proliferating cell nuclear antigen, Cat. 13,110), FASN (fatty acid synthase, Cat. 3,180), P-AKT (phosphorylation of protein kinase B, Cat. 4,060), γ-H2AX (gamma-Histone H2AX, Cat. 9,718), and P-NFκB (phosphorylation of nuclear factor kappa-B, Cat. 3,033) and GAPDH (glyceraldehyde-3-phosphatedehydrogenase, Cat. 5,174) were obtained from Cell Signaling Technology (Boston, United States).

### Cell culture

The C18-4 cell line was established by transfection with mouse SSCs. C18-4 cells possess phenotypic characteristics that are similar to SSCs, as evidenced by the expressions of various SSC markers, including OCT-4, GFRA1, and PLZF (25). The C18-4 cells were cultured with DMEM/F12 medium containing 10% FBS (Gibco, Grand Island, United States), 2 mM glutamine, and 1% penicillin–streptomycin at 37°C in humidified air containing 5% of CO_2_. The cells were passaged when they reached 80% confluency.

### Animals

C57BL/6 mice (5–6 weeks old) were obtained from Hunan SJA Laboratory Animal Co., Ltd. (Changsha, Hunan, China) and housed in a specific pathogen-free (SPF) animal facility. This study was approved by the Biomedical Research Ethics Committee, School of Medicine, Hunan Normal University (Reference: D2021036). The mice were fed in individually ventilated cages with sufficient and accessible food and water in a standard environment required for laboratory animal breeding. The animals were divided into four groups (*n* = 6, each group) with different treatments: vehicle, intraperitoneal injection of PTC-209 (20 mg/kg, a representative inhibitor of BMI1), intragastric administration of α-tocopherol (8 mg/kg), and the combination of PTC-209 and α-tocopherol. All treatments were given five times/week for 3 weeks. Drug dissolution details are as follows: vehicle: 5% Tween 80 in sterilized water; PTC-209: 5% PTC-209 stock solution +40% PEG300 + 5% Tween 80 + 50% sterilized water; α-tocopherol: stock solution diluted to the desired concentration with sterilized water.

### Methyl thiazolyl tetrazolium (MTT) assay

Cell proliferation was detected using the MTT assay. Mouse SSCs (C18-4 cells) were seeded into 96-well microplates (Thermo Fisher Scientific, Waltham, MA, United States) containing 200 μL of culture medium at a density of 6,000 cells/well. After cell attachment, the cells were treated with various concentrations of α-tocopherol (0–128 μg/mL; the molecular weight of α-tocopherol is 430.71; thus 128 μg/mL of α-tocopherol is equivalent to 297 μM) transfected with the control siRNA (siCtrl), BMI1 siRNA1 (siBMI1-1), BMI1 siRNA2 (siBMI1-2), BMI1 siRNA3 (siBMI1-3), or in combination with α-tocopherol (32 μg/mL = 74.25 μM). After 3 days of culture, the tetrazolium salt of MTT (50 μL; Sigma-Aldrich, Shanghai, China) was dissolved in PBS to a concentration of 2 mg/mL and then added to each well. The plates were then incubated for another 5 h. Next, the medium was aspirated from each well, and 150 μL of DMSO was added to dissolve formazan crystals. A microplate reader (Biotek, SYNERGY HTX, Vermont, United States) was used to measure the absorbance (OD values) of each well at 490 nm.

### Immunofluorescence (IF)

For IF, C18-4 cells were incubated in 6-well plates attached to a glass slide. Cells were treated with various concentrations of α-tocopherol (0–128 μg/mL), transfected with siCtrl, siBMI1-1, siBMI1-2, and siBMI1-3, or in combination with α-tocopherol (32 μg/mL). After harvesting, the cells were fixed with 4% paraformaldehyde for 15 min at room temperature and washed three times with 1× PBS (phosphate-buffered saline). Then, cells were permeabilized with 0.1% Triton for 10 min and then blocked with 5% bovine serum and incubated overnight at 4°C with the indicated primary antibody. On the second day, coverslips were washed three times with PBS, incubated with an appropriate secondary antibody for 45 min at room temperature, and then stained with 4, 6-diamidino-2-phenylindole (DAPI). A light microscope (Leica, DM3000, Germany) was used to capture immunofluorescent images.

For tissue IF staining, frozen slides were prepared and baked at 37°C for 10–20 min to remove moisture, and then fixed in 4% paraformaldehyde for 30 min. Antigen retrieval was performed using 1 mM EDTA unmasking solution (Servicebio, Wuhan, China) and microwave heating. After cooling down, the slides were blocked with PBS/5% BSA for 1 h, and then incubated overnight at 4°C with the indicated primary antibody diluted in PBS/1% BSA. After washing, the slides were incubated with Alexa Fluor-conjugated secondary antibodies (Invitrogen, Carlsbad, Unnited States) at room temperature in the dark for 1 h, after which they were counterstained with DAPI solution for 10 min, and stored in the dark. Images were captured using a fluorescence microscope (Leica, DM3000, Germany).

### RNA isolation and quantitative reverse transcription (RT) PCR

Total RNA was extracted from C18-4 cells with Trizol reagent after various treatments. A reverse transcription (RT) kit (Vazyme, Nanjing, China) was used for the reverse transcription of mRNA into cDNA at 42°C for 2 min with 0.6 μg of total RNA, 4 μl of 4× gDNA wiper mix, and 10 μL of RNase-free water. Then, 4 μL of 5× HiScriptII qRT SuperMixII was added to the reaction mixture. The reaction was continued for 15 min at 50°C and 5 s at 85°C. The mRNA primer sequences for real-time PCR are shown in Supplementary Table S1. Real-time PCR was performed three times with a CFX Connect Real-Time System (BIORAD, Hercules, United States) according to the manufacturer’s instructions.

### Small interfering RNA (siRNA) transfection

C18-4 cells were transfected with commercially available BMI1 siRNAs with the transfection reagent Lipofectamine 6,000 (Invitrogen, Carlsbad, USA). In brief, cells were plated in 6-well plates at a density of 3 × 10^5^ cells per dish; cells at 30–50% confluence were first transfected with 50 nM BMI1 siRNA, or 50 nM negative control siRNA using Lipofectamine 6,000 in the absence of antibiotics, or FBS for 6 h. After infection, the cells were washed twice with 1× PBS and supplemented with DMEM/F12 medium for the indicated time. Cell protein was collected, and specific silencing was confirmed by western blotting. The targeting sequences were follows: siBMI1-1: GCCACTACCATAATAGAAT; siBMI1-2: GGACATTGCCTACATTTAT; siBMI1-3: CCATGAATGGAACCAGCAA.

### Western blotting

C18-4 cells were incubated in 6-well plates at a density of 5 × 10^5^ cells/well. Following incubation, the cells were washed twice with cold PBS and lysed in lysis buffer. The lysate was then sonicated, incubated on ice for 30 min and centrifuged. Total protein concentration in the supernatant was measured using the BCA protein assay kit (Solarbio, Beijing, China). For tissue samples, an appropriate amount of tissue was homogenized using Tissuelyser (Servicebio, Wuhan, China) in ice-cold lysis buffer. The lysate was then centrifuged at 13000 rpm for 10 min at 4°C; then, pelleted tissue debris was removed. The supernatant was sonicated, incubated on ice for 30 min and centrifuged. Total protein concentration was assessed using the BCA protein assay kit. An equal amount of total protein was loaded onto SDS-PAGE gels. The proportion of acrylamide/bis acrylamide used depended on the molecular weight of the determined protein. Proteins were then transferred onto polyvinylidene difluoride (PVDF) membranes (Millipore, Boston, United States) which were then blocked in 5% skimmed milk in TBST at room temperature for 1 h, incubated with the indicated primary antibodies overnight at 4°C, and then incubated with a suitable secondary antibody for 1 h at room temperature. Finally, the bands were detected using ChemiDoc Touch (BIORAD, Hercules, United States) with ClarityTM Western ECL Substrate (BIORAD, Hercules, United States) and quantified using Image J (NIH, Baltimore, United States).

### 5-ethynyl-2′-deoxyuridine (EdU) incorporation assays

For Edu assays, 6,000 C18-4 cells/well were plated into 96-well plates, treated with various concentrations of α-tocopherol (0–128 μg/mL), and transfected with siCtrl, siBMI1-1, siBMI1-2, siBMI1-3, or in combination with α-tocopherol (32 μg/mL). Subsequently, 20 μM 5-ethynyl-2′-deoxyuridine (EdU, ApexBio, Houston, TX, United States) was added to the cells and incubated with DMEM/F12 for 12 h. The medium was then removed, and the cells were fixed with 4% paraformaldehyde for 20 min and permeabilized with 0.5% Triton X-100 for 10 min. Immunofluorescence staining was then performed to detect EdU-positive cells using anti-EdU. Hoechst 33342 was applied to label the cell nuclei, and images were captured by a fluorescence microscope (Leica, DM3000, Germany).

### Sperm count

The left seminiferous tubule of each experimental mouse was removed and placed into a petri dish containing 1.5 mL of culture medium and incubated at 37°C for 15 min; this allowed sperm in the tubule to be released into the culture medium. The culture medium containing sperm was then mixed and centrifuged at 200 g for 5 min. The supernatant was removed and 200 μL of culture medium was added to the tube and mixed well. Next, 10 μL of the sperm-containing mixture was then smeared onto slides and counted using a microscope (Leica, DM3000, Germany).

### Sperm staining

For staining, 10 μL of the sperm-containing mixture was smeared onto slides and dyed with 0.5% gentian purple alcohol solution for 10 min. The slides were then washed and the sperm morphology was observed under a microscope to determine normality (Leica, DM3000, Germany).

### Statistical analysis

All data are presented as mean ± standard deviation (SD) of at least three independent experiments. Differences between the two groups were assessed for significance using the Student’s *t*-test. Multiple group comparisons were analyzed by one-way analysis of variance (ANOVA). Graphs were generated by GraphPad Prism 9.4 (GraphPad Software Inc., San Diego, CA, United States). Differences of *p < 0.05 was considered statistically significant (*p < 0.05, **p < 0.01, ***p < 0.001, ****p < 0.0001).

## Results

### BMI1 was expressed at high levels in mouse testis and spermatogonia

**Figure 1:**
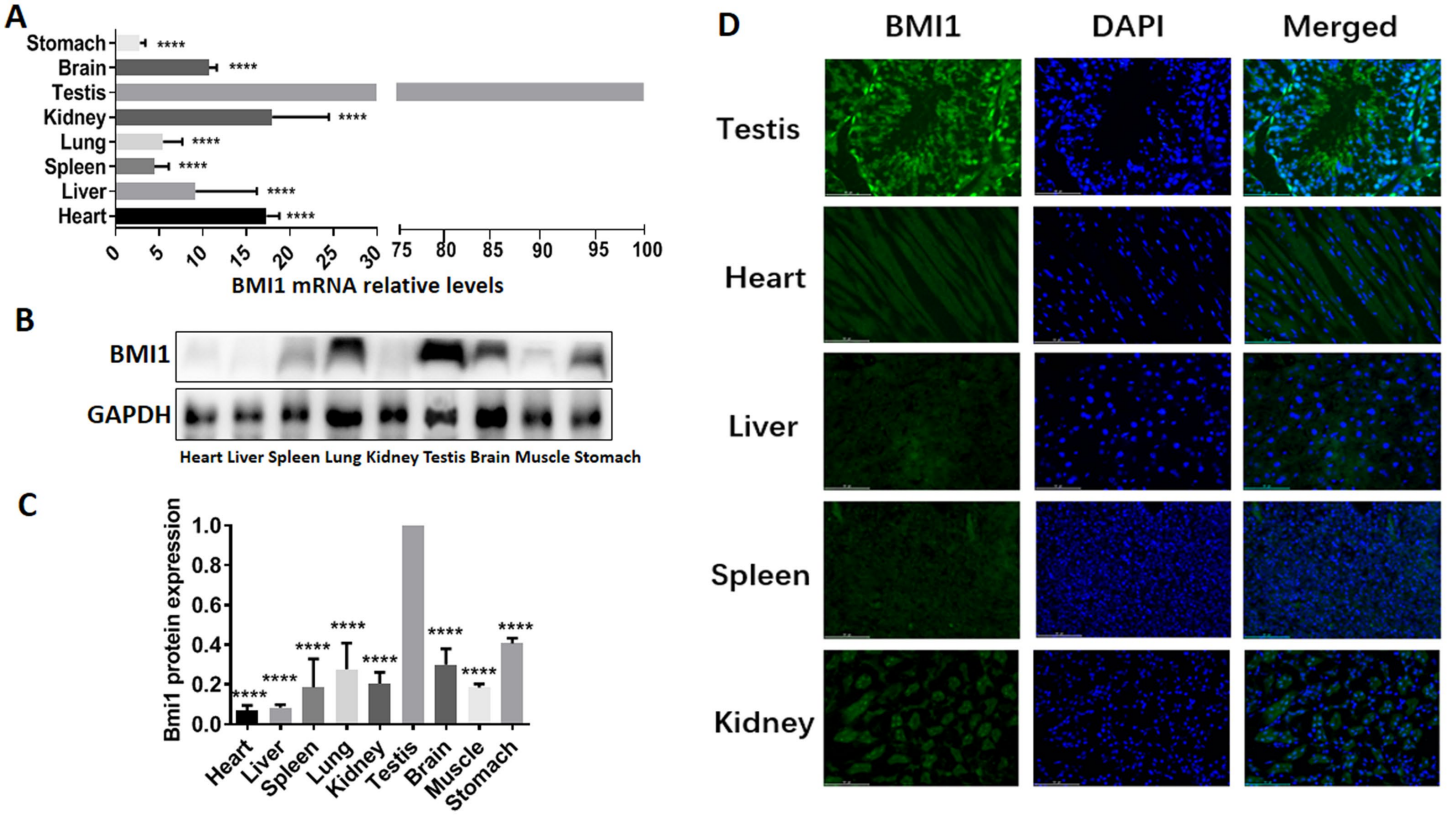
BMI1 was expressed at high levels in mouse testis and spermatogonia.

To determine whether there was a correlation between BMI1 and the reproductive system, we detected the gene and protein expression of BMI1 in various mouse tissues by real time-PCR, western blotting, and immunofluorescence. Higher levels of BMI1 mRNA (*p* < 0.0001) and protein (*p* < 0.0001) were detected in mouse testis than in other tissues (Figures 1A-D, Supplementary Figure S1). Notably, BMI1 was expressed mainly in the spermatogonia along the basement membrane of the seminiferous tubules of mouse testis (Figure 1D). Collectively, these findings indicated that BMI1 is expressed at high levels in mouse testis and spermatogonia.

### BMI1 Silencing inhibited the proliferation and DNA synthesis of mouse SSCs

**Figure 2:**
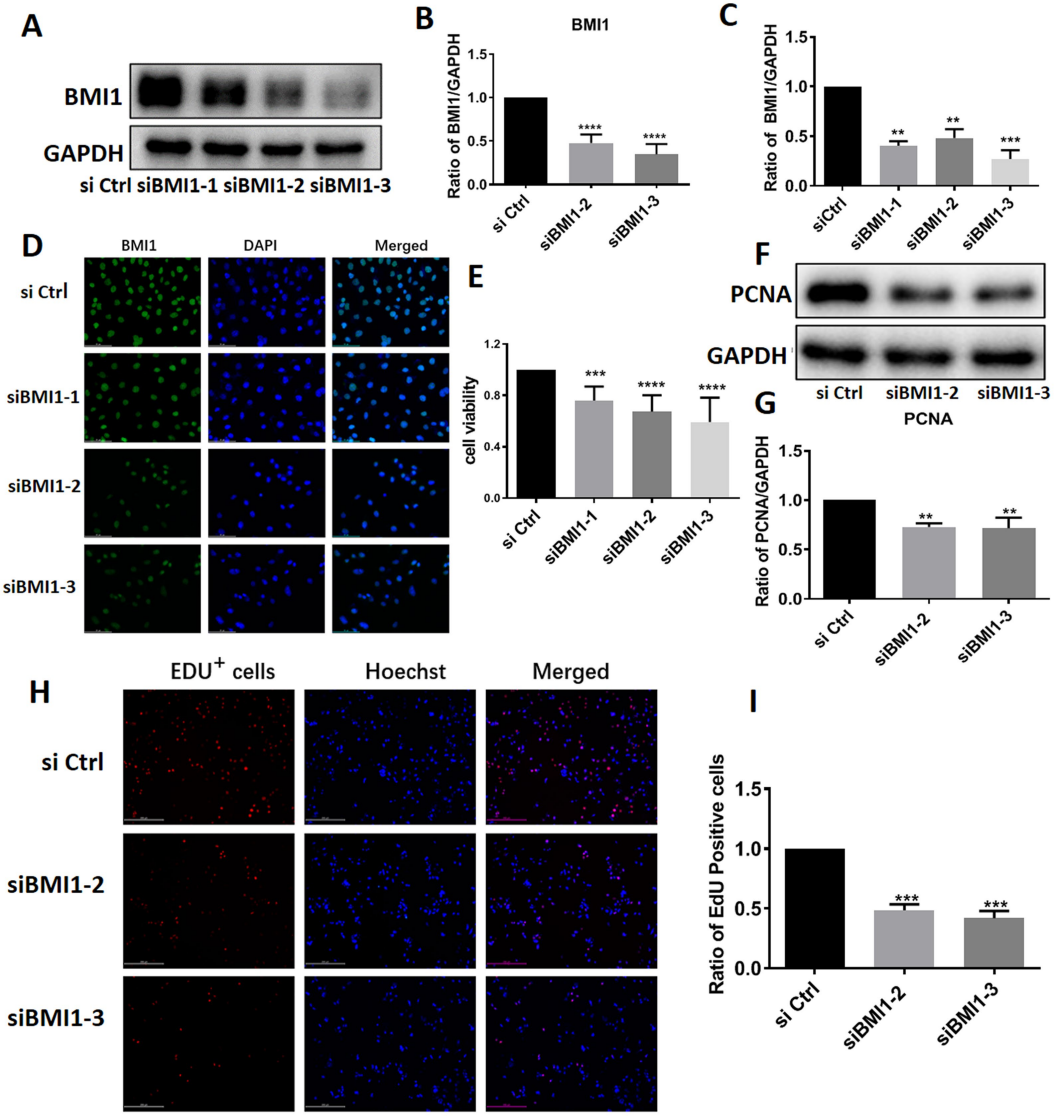
BMI1 silencing suppressed the proliferation and DNA synthesis of mouse SSCs.

To investigate the effect of BMI1 silencing on mouse SSC proliferation, we used four siRNA sequences: control siRNA (siCtrl), BMI1 siRNA-1, −2, and − 3 (siBMI1-1, siBMI1-2, siBMI1-3, respectively). We demonstrated that siBMI1-2 and siBMI1-3 effectively knocked down the mRNA and protein levels of BMI1 in mouse SSCs (Figures 2A-D). Therefore, we chose siBMI1-2 and siBMI1-3 for subsequent experiments.

The effect of *BMI1* silencing on the cell proliferation of mouse SSCs was detected by various approaches. MTT assays revealed that cell viability was significantly reduced in C18-4 cells following the transfection of siBMI1-2 (*p* < 0.0001) and siBMI1-3 (*p* < 0.0001) (Figure 2E). Notably, the levels of proliferating cell nuclear antigen (PCNA), a cell proliferation marker, were reduced in C18-4 cells, as detected by siBMI1-2 (*p* = 0.0041) and siBMI1-3 (*p* = 0.0033) (Figures 2F,G). EdU incorporation assays showed that siBMI1-2 (*p* = 0.001) and siBMI1-3 (*p* = 0.001) significantly reduced the proportion of EdU-positive cells in C18-4 cells (Figures 2H,I). These findings show that BMI1 silencing inhibited the proliferation and DNA synthesis of mouse SSCs.

### BMI1 Silencing decreased phos-AKT and increased DNA damage in mouse SSCs

**Figure 3:**
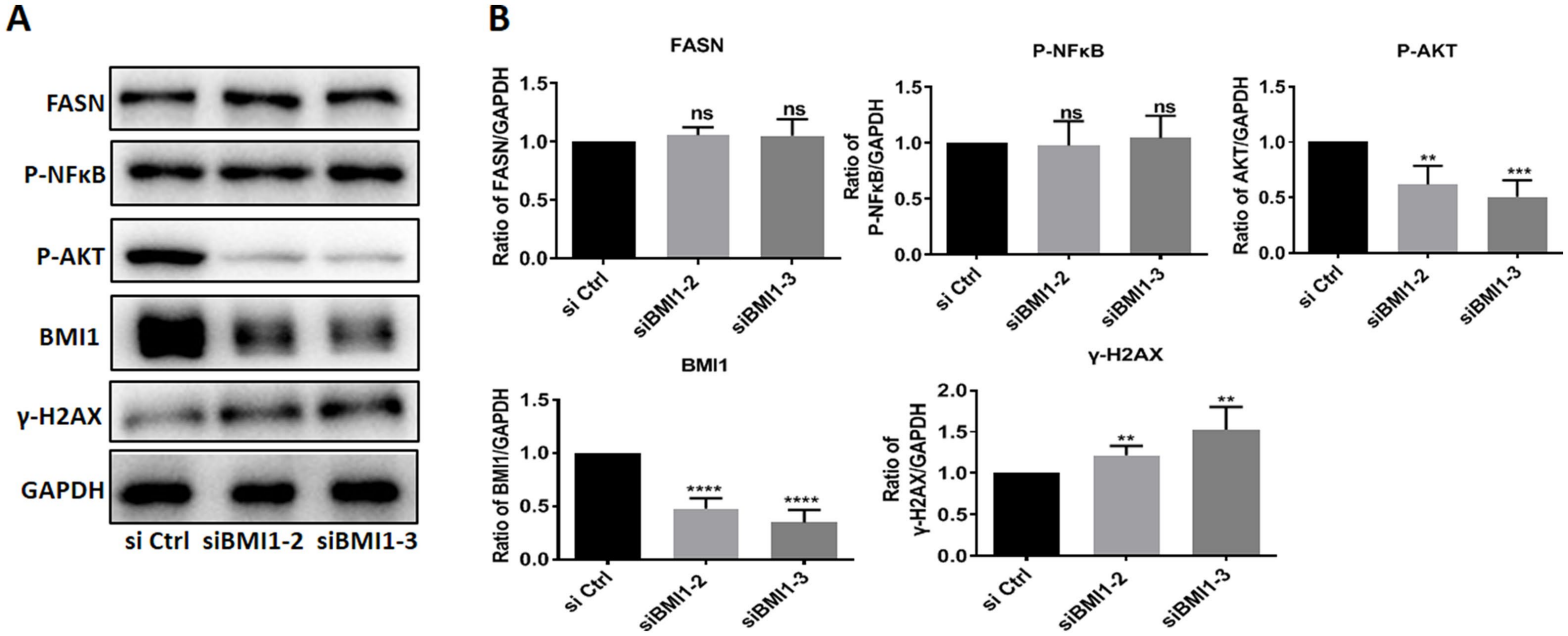
BMI1 silencing reduced the levels of phos-AKT and increased the levels of γ-H2AX in mouse SSCs.

Next, we investigated whether BMI1 silencing affected the signaling pathways in mouse SSCs. The knockdown of BMI1 suppressed the expression levels of phos-AKT but increased the levels of γ-H2AX, a marker of DNA damage, in C18-4 cells (Figures 3A,B). There were no changes observed in the expression levels of FASN and phos-NFκB (Figures 3A,B). Our findings indicate that BMI1 silencing deactivated the AKT pathway and caused DNA damage in mouse SSCs.

### Alpha-tocopherol up-regulated BMI1 expression and promoted the proliferation of mouse SSCs

**Figure 4:**
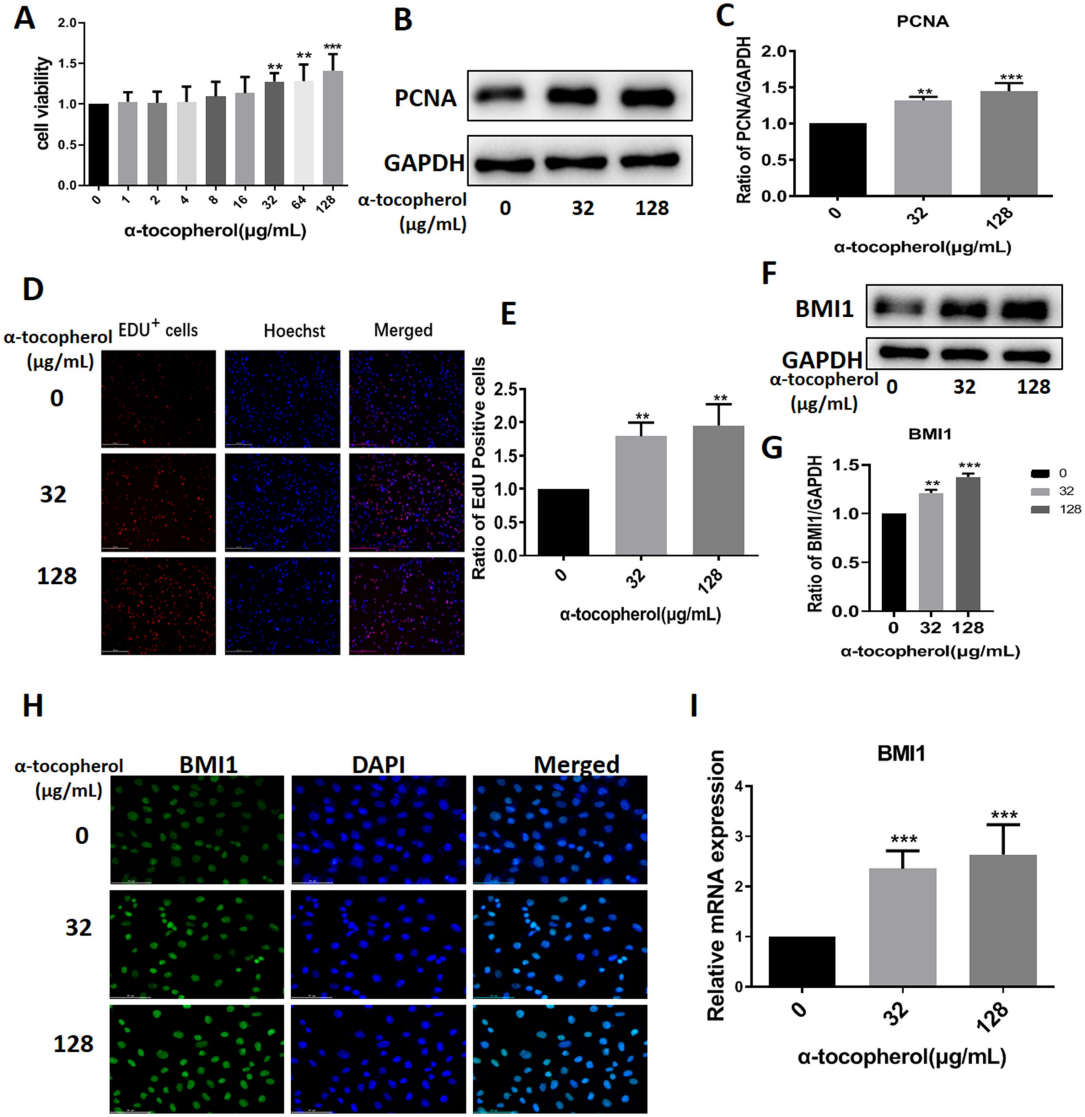
α-tocopherol promoted mouse SSC proliferation and up-regulated BMI1 expression.

We screened several available compounds, including α-tocopherol, metformin, betaine, and zinc chelate (data not shown), which may promote mouse SSC proliferation. Notably, we found that α-tocopherol promoted the proliferation of C18-4 cells at concentrations ranging from 32 to 128 μg/mL (Figure 4A). Interestingly, α-tocopherol treatment significantly increased the levels of PCNA protein (0 vs. 32 μg/mL, *p* = 0.0027; 0 vs. 128, *p* = 0.0005) in C18-4 cells (Figures 4B,C). Furthermore, α-tocopherol significantly increased the proportion of EdU-positive (0 vs. 32 μg/mL, *p* = 0.0079; 0 vs. 128 μg/mL, *p* = 0.0032) C18-4 cells, thus indicating that α-tocopherol promotes mouse SSC proliferation and DNA synthesis (Figures 4D,E). Importantly, α-tocopherol, at concentrations of either 32 μg/mL or 128 μg/mL, enhanced the protein (0 vs. 32 μg/mL, *p* = 0.0002; 0 vs. 128 μg/mL, *p* < 0.0001) and mRNA (0 vs. 32 μg/mL, *p* = 0.0007; 0 vs. 128 μg/mL, *p* = 0.0001) levels of BMI1 in C18-4 cells (Figures 4F-G,I). Immunofluorescence further revealed that the levels of BMI1 protein increased in C18-4 cells following α-tocopherol treatment (Figure 4H). Therefore, these findings demonstrate that α-tocopherol promoted mouse SSC proliferation and DNA synthesis by up-regulating BMI1 expression.

### Alpha-tocopherol restored the reduction of proliferative ability In mouse SSCs caused by BMI1 silencing

**Figure 5:**
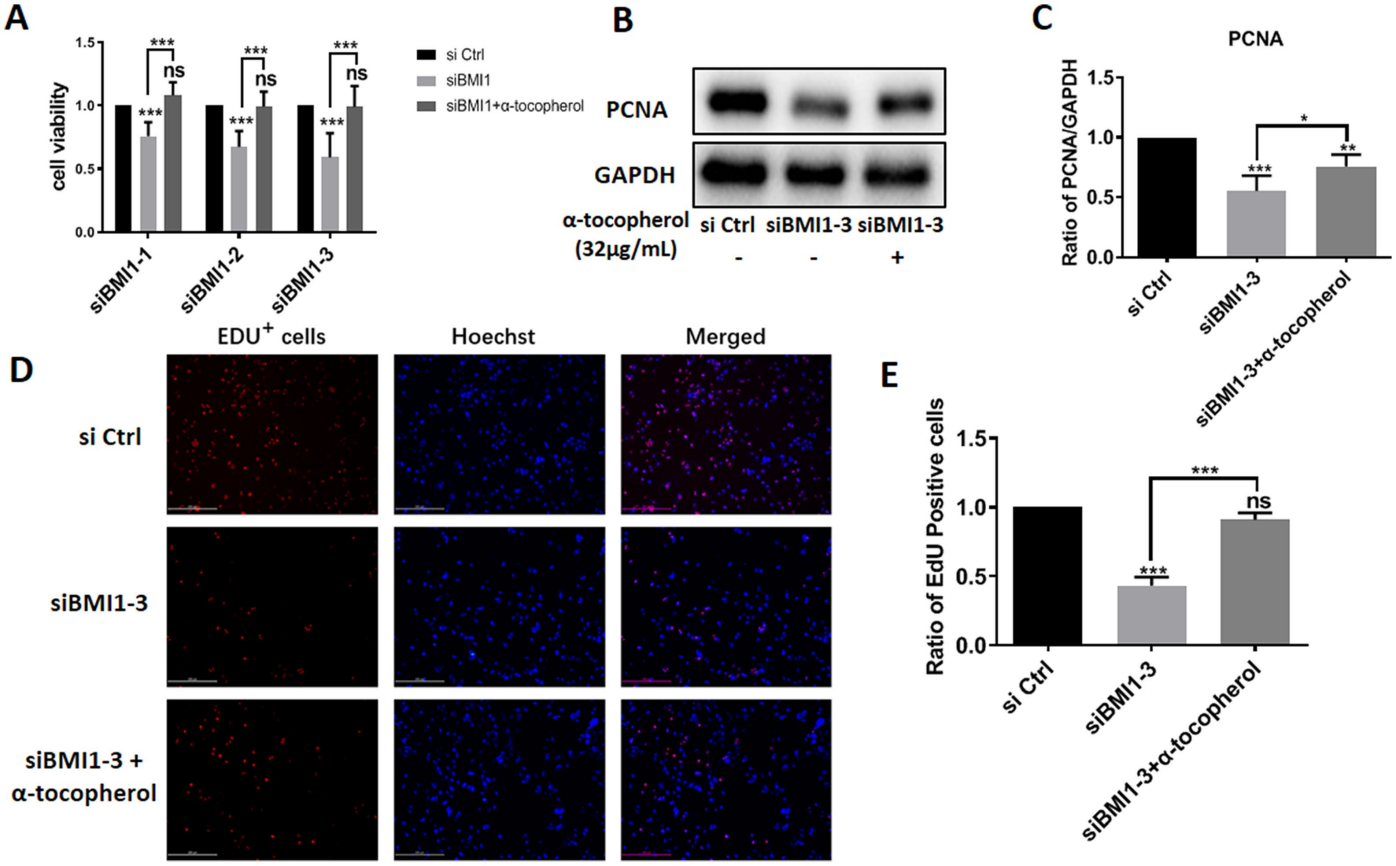
α-tocopherol restored the reduced proliferation and DNA synthesis of mouse SSCs caused by BMI1 silencing.

Next, we investigated whether α-tocopherol had any effect on the reduction of proliferation in mouse SSCs caused by BMI1 silencing. MTT assays revealed that α-tocopherol (32 μg/mL) restored the reduced proliferative ability of C18-4 cells after BMI1 silencing (Figure 5A). Furthermore, α-tocopherol rescued the reduced levels of PCNA in C18-4 cells caused by siBMI1-3 (Figures 5B,C). This effect of α-tocopherol on DNA synthesis was further demonstrated by EdU incorporation assays, in that the ratio of EdU-positive cells was reduced after silencing BMI1, whereas α-tocopherol restored this ratio to its normal level (Figures 5D,E). Taken together, these findings suggest that α-tocopherol restored the reduction in proliferation and DNA synthesis of mouse SSCs caused by BMI1 silencing.

### Alpha-tocopherol attenuated the effect of BMI1 silencing-induced alterations of phos-AKT and DNA damage in mouse SSCs

**Figure 6:**
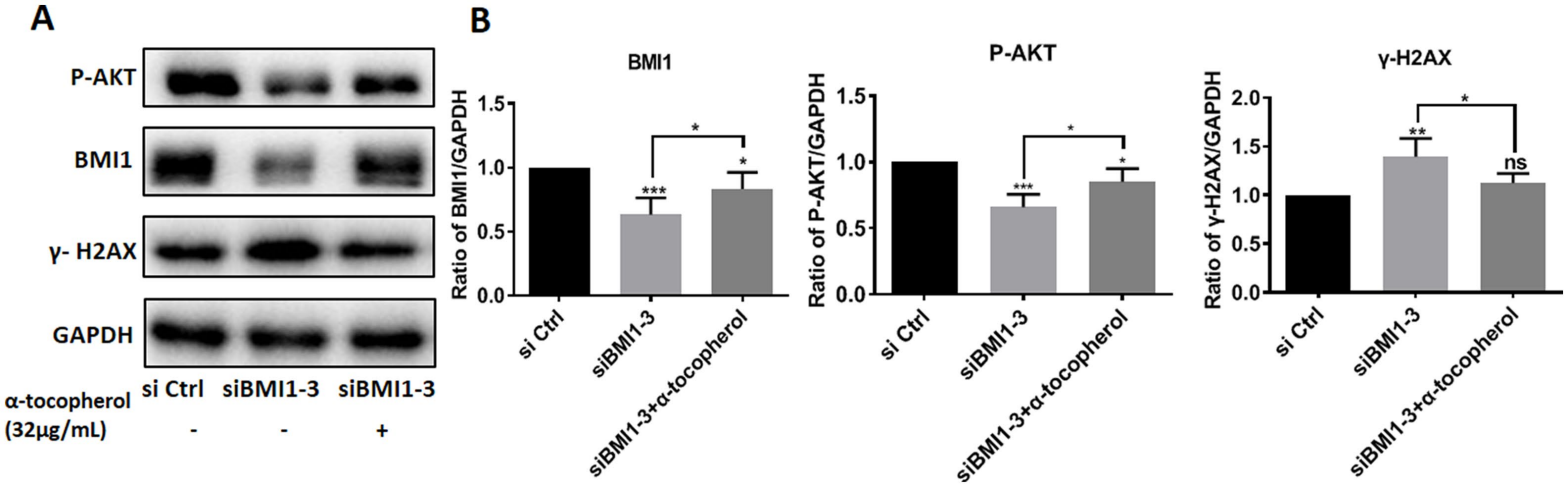
The efficacy of α-tocopherol on phos-AKT level and DNA damage in mouse SSCs caused by BMI1 silencing.

To investigate whether adding α-tocopherol ameliorated BMI1 silencing-induced alterations of protein levels, we treated C18-4 cells with α-tocopherol (32 μg/mL) for 24 h after transfecting with siBMI1-3. α-tocopherol increased the phos-AKT reduction caused by BMI1 silencing but reduced the level of γ-H2AX caused by BMI1 silencing (Figures 6A,B). These findings imply that α-tocopherol is involved in the AKT signaling pathway and DNA damage of mouse SSCs and is regulated by BMI1.

### Alpha-tocopherol restored mouse sperm malformation caused By BMI1 inhibitor *in vivo*

**Figure 7:**
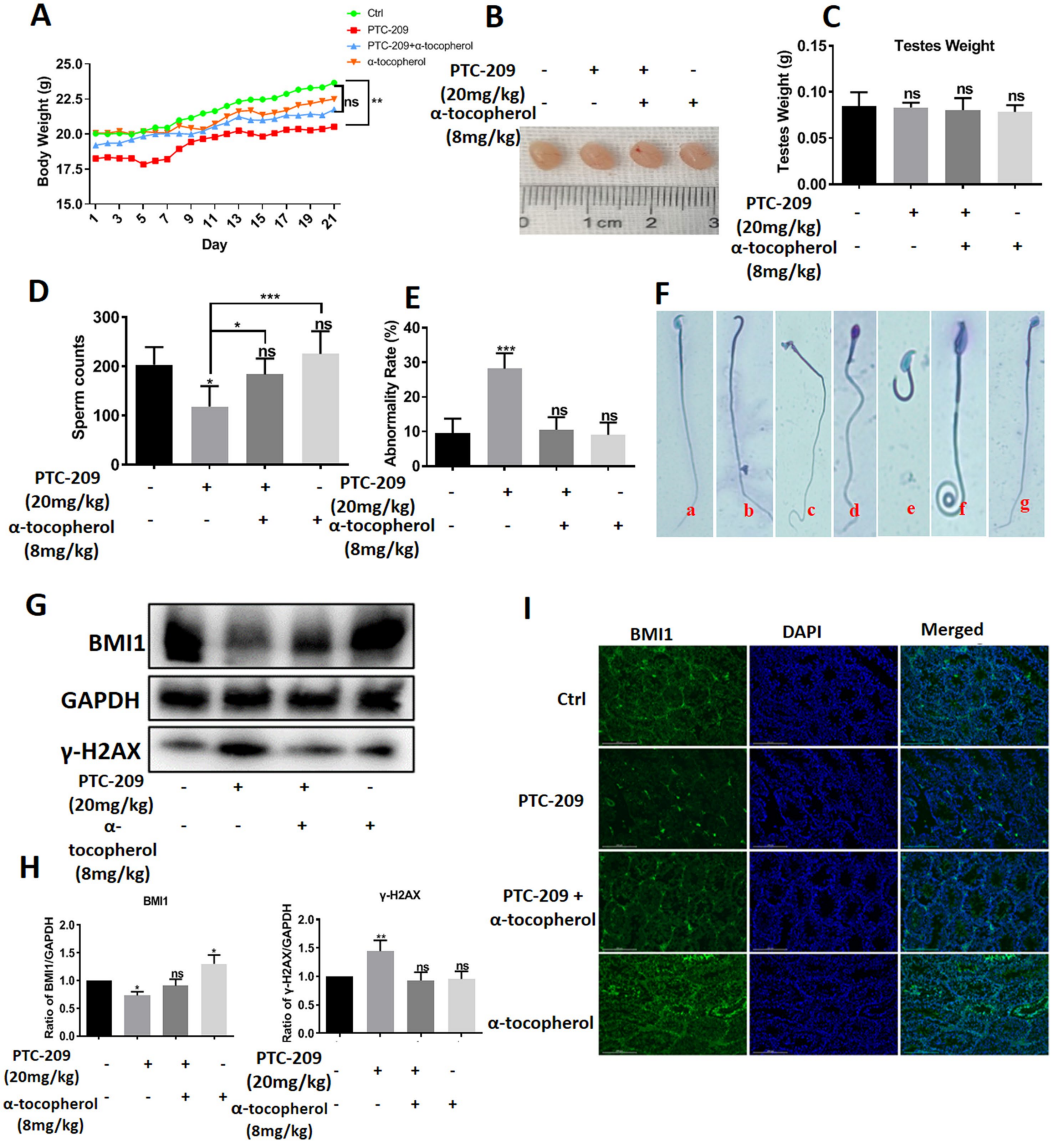
α-tocopherol restored the mouse spermatid malformations caused by BMI1 inhibitor.

To investigate the function of BMI1 and α-tocopherol in controlling the morphology of spermatids, we randomly classified mice into four groups: a Ctrl group, PTC-209 group, PTC-209 + α-tocopherol group, and α-tocopherol group. The doses of PTC-209 and α-tocopherol were 20 and 8 mg/kg, respectively. PTC-209 is a specific inhibitor of BMI1. The weight of each mouse was recorded daily. After five times per week treatments for 3 weeks, spermatids in the left vas deferens of mice were counted and stained to measure the rate of spermatid malformation. As shown in Figure 7A, the body weight of mice in the PTC-209 group was significantly lower than that in the Ctrl group (*p* = 0.009), whereas the body weight of mice in the other two groups were not significantly different when compared to the Ctrl group. The size and weight of testes in the four groups were similar (Figures 7B,C). However, the sperm count of mice in the PTC-209 group was significantly lower than that in the Ctrl group (*p* = 0.034). Notably, the sperm count of mice in the combined group (PTC-209 + α-tocopherol) was close to normal. Furthermore, the sperm count of mice in the α-tocopherol group was a slightly higher than that of mice in the Ctrl group, although this was not statistically significant (Figure 7D). Furthermore, there were no alterations in sperm morphology as shown in (Figure 7E), the abnormality rate of sperm was remarkably higher in the PTC-209 group (*p* < 0.001). We observed numerous defects in the sperm head, including the absence of heads, broken heads, and enlarged and irregular heads. We also observed sperm tail defects, including lost and curled tails (Figure 7F). Notably, PTC-209-induced abnormality of sperms was reversed into normal status in the combined group. These results suggested that α-tocopherol could improve the malformations in mouse spermatids induced by the BMI1 inhibitor. To further investigate the association between α-tocopherol and BMI1, we measured the protein alterations of testes in the four groups. As shown in (Figures 7G,H), the BMI1 inhibitor PTC-209 reduced the level of BMI1 protein expression in the testis (*p* = 0.0351); however, this reduction was restored by the combination of α-tocopherol and PTC-209.

Furthermore, α-tocopherol alone led to an increase in BMI1 expression in a manner that was consistent with the fact that BMI1 levels in C18-4 cells were changed by α-tocopherol *in vitro*. The BMI1 IF levels in the testis of mice in the four groups showed consistent results (Figure 7I). Furthermore, the levels of γ-H2AX in mouse testis were significantly enhanced with PTC-209 (*p* = 0.0099); however, this level was reduced by combining α-tocopherol with PTC-209 (Figures 7G,H). Taken together, our results demonstrate that α-tocopherol mediated BMI1 expression in mouse testis *in vivo*.

## Discussion

Male mice with BMI1 deficiency exhibit severe oligospermia and complete infertility (14), thus suggesting that BMI1 plays an important role in maintaining normal fertility in males. In the present study, we demonstrated that BMI1 was expressed at high levels in mouse testis at both gene and protein levels; these findings are with the findings of a previous report (26). We also found that BMI1 was expressed in other organs, including the kidney, heart, liver, and brain, but to a much lesser extent than in the testis. BMI1 is present in almost all types of human tissues, with the highest expression in the testis, and is considered a multifunctional regulatory factor involved in various biological processes, especially the maintenance of stem cell growth (9, 27, 28). Analysis and comparison of BMI1 nucleotide and deduced amino acid sequences between mice and humans revealed high levels of homology (29). Thus, our present study focused on the role of BMI1 on mouse SCC proliferation. To investigate the function of BMI1 in controlling mouse SSC growth, we used three siRNA sequences to silence BMI1 in mouse SSCs. MTT and EdU incorporation assays revealed that BMI1 silencing inhibited the proliferation of mouse SSCs. The reduced levels of PCNA protein suggested that BMI1 silencing blocked DNA synthesis in mouse SSCs. Furthermore, we found that silencing BMI1 reduced the expression levels of phos-AKT, a protein closely associated with cell proliferation. The levels of γ-H2AX, a hallmark of DNA damage, was increased in C18-4 cells after BMI1 silencing, thus implying that BMI1 knockdown can cause DNA damage. Collectively, these findings implicate that BMI1 silencing suppressed the proliferation and DNA synthesis of mouse SSCs and increases DNA damage in these cells.

Alpha-tocopherol, delivered through hybrid carbohydrate-antioxidant polymers to porcine spermatozoa, can reduce oxidative damage in sperm and improve fertility (30). The mixture of α-tocopherol, L-ascorbic acid, zinc, and selenium can promote chemotherapeutic drug-induced germ cell proliferation and protect sperm structure and function (31). However, the effect of α-tocopherol on SSCs and the mechanisms involved have yet to be elucidated. In this study, we demonstrated that α-tocopherol not only promoted the proliferation of mouse SSCs but also up-regulated the expression level of BMI1, a key protein that regulates cell proliferation and differentiation. We also found that α-tocopherol recovered the reduction of proliferation in mouse SSCs induced by BMI1 silencing and restored the expression levels of BMI1, phos-AKT, and γ-H2AX to normal levels. Together, these findings demonstrate that α-tocopherol promoted the proliferation of mouse SSCs by regulating the BMI1 and AKT pathways.

The functions of BMI1 and α-tocopherol *in vivo* were further investigated in sexually mature male mice. Previous studies have demonstrated that deleting the *BMI1* gene leads to severe azoospermia, reduced testicular weight, and infertility in male mice (14, 32). We demonstrated that the inhibition of BMI1 reduced sperm count and severe sperm malformation in mice, thus indicating the importance of BMI1 in maintaining male reproduction. However, we did not observe a reduction in testicular weight in the present study when using PTC-209, a representative inhibitor of BMI1. This may have been because the knockout of BMI1 completely removed BMI1 expression, whereas PTC-209 treatment left BMI1 expression partially intact. Notably, the combination of α-tocopherol and PTC-209 was shown to restore sperm count. We detected alterations in BMI1 protein levels in testicular tissues subjected to various treatment conditions by applying Western blotting and IF techniques. Analysis consistently revealed that PTC-209 significantly reduced the level of BMI1 while α-tocopherol enhanced its expression. Furthermore, α-tocopherol alleviated PTC-209-reduced BMI1 protein expression. These findings suggest that α-tocopherol exerted its protection on mouse sperm by up-regulating BMI1. We also found that α-tocopherol reduced the significant increase in the expression of γ-H2AX in response to PTC-209, thus indicating that α-tocopherol reduced PTC-209-induced DNA damage in the testis. Based on our *in vitro* and *in vivo* data, we can conclude that we have demonstrated that BMI1 controls male infertility. It is worth noting the biological function of α-tocopherol *in vivo*, which almost fully restored the sperm count and sperm malformation rates caused by treatment with a BMI1 inhibitor. Therefore, the present study demonstrated that α-tocopherol has potential applications in maintaining and treating male infertility.

## Conclusion

In the present study, we demonstrated that BMI1 was expressed at high levels in mouse testicular tissue and spermatogonia and that BMI1 was essential for mediating the proliferation and DNA synthesis of mouse SSCs. BMI1 silencing inhibited the proliferation and DNA synthesis of mouse SSCs and enhanced DNA damage in these cells. Notably, we found that α-tocopherol could restore the proliferation and DNA damage of mouse SSCs *in vitro*, as well as the sperm count and sperm malformation *in vivo* caused by BMI1 deficiency. Therefore, our findings shed new insights into the molecular mechanisms underlying the fate of mammalian SSCs and might offer novel targets and approaches for treating male infertility.

## Data availability statement

The original contributions presented in the study are included in the article/Supplementary material, further inquiries can be directed to the corresponding authors.

## Ethics statement

The animal study was reviewed and approved by Biomedical Research Ethics Committee, School of Medicine, Hunan Normal University, serial number D2021036. Written informed consent was obtained from the owners for the participation of their animals in this study.

## Author contributions

MP and JW finished the main part of the experiments and were major contributors in writing the manuscript. WW and SX participated in animal processing of animal samples. TL and DX drafted the experimental scheme. ZH and XY revised the manuscript. All authors contributed to the article and approved the submitted version.

## Funding

This project was supported by Institutional Open Fund (KF2022001 and KF2021017), Key Project of Developmental Biology and Breeding from Hunan Province (2022XKQ0205), Scientific and Technological Projects for Collaborative Prevention and Control of Birth Defect in Hunan Province (2019SK1012), and Key Grant of Research and Development in Hunan Province (2020DK2002).

## Conflict of interest

The authors declare that the research was conducted in the absence of any commercial or financial relationships that could be construed as a potential conflict of interest.

## Publisher’s note

All claims expressed in this article are solely those of the authors and do not necessarily represent those of their affiliated organizations, or those of the publisher, the editors and the reviewers. Any product that may be evaluated in this article, or claim that may be made by its manufacturer, is not guaranteed or endorsed by the publisher.
