# Supplementary material for: Alpha-tocopherol enhances spermatogonial stem cell proliferation and restores mouse spermatogenesis by up-regulating BMI1
Source: Front Nutr. 2023 Apr 17;10:1141964. doi: 10.3389/fnut.2023.1141964 (PMC10150882; doi:10.3389/fnut.2023.1141964)
Supplement: Supplementary file 1 [file Table_1.DOCX]

Table S1. The primer sequences for Real-time Reverse Transcription (RT) PCR

| Gene | Forward | Reverse |
| --- | --- | --- |
| BMI1 | AATGAAGATGAGTCACCAGAGG | CAAAGGAAGATTGGTGGTTAGC |
| GAPDH | AACCCAAACTAACAGTTGTCCCAA | ACTCCTTGGAGGCCATGTAGG |
