# Supplementary figures and images for: Alpha-tocopherol enhances spermatogonial stem cell proliferation and restores mouse spermatogenesis by up-regulating BMI1
Source: Front Nutr. 2023 Apr 17;10:1141964. doi: 10.3389/fnut.2023.1141964 (PMC10150882; doi:10.3389/fnut.2023.1141964)

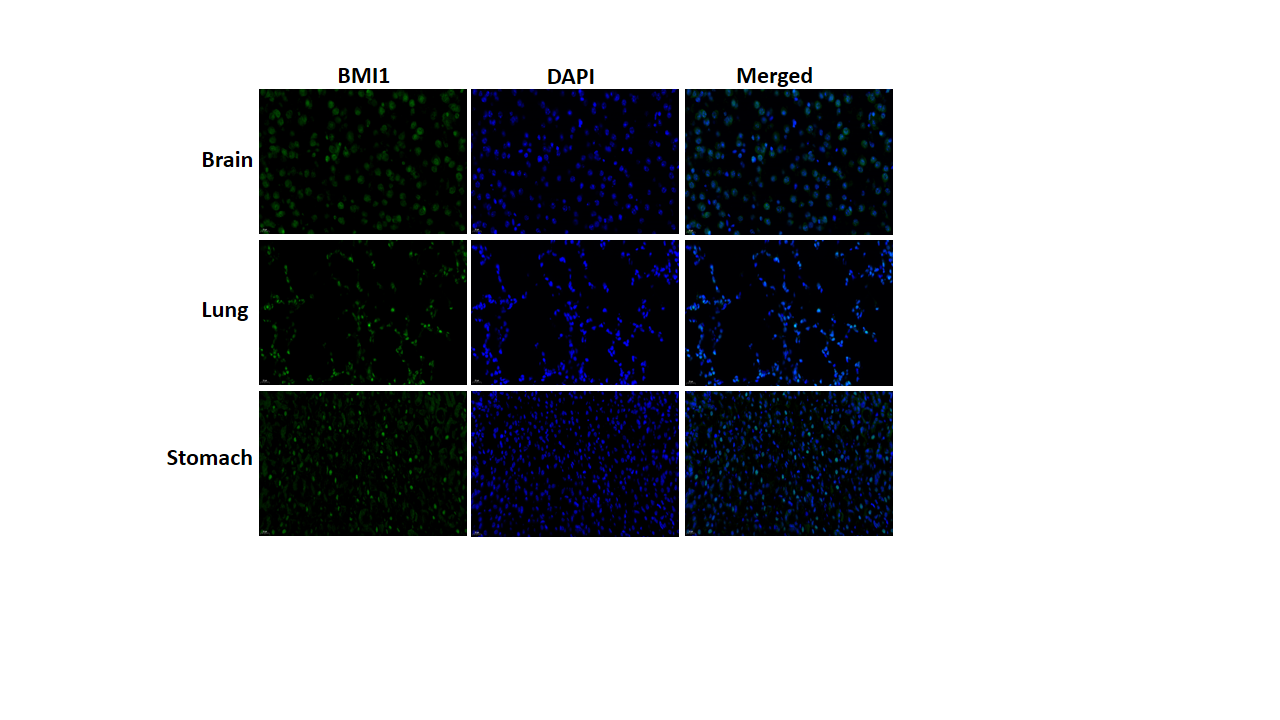

Supplement: Supplementary file 2 [file Image_1.TIF]
